# Retraction: Questioning the HIV-AIDS hypothesis: 30 years of dissent

**DOI:** 10.3389/fpubh.2019.00334

**Published:** 2019-10-29

**Authors:** Frontiers Editorial Office

In 2014, *Frontiers in Public Health* published an article by Dr. Patricia Goodson, Texas A&M University: “Questioning the HIV-AIDS hypothesis: 30 years of dissent”.

The article was submitted under our “Hypothesis and Theory” article-type and purported to review “the most salient questions raised, alongside theories proposing non-viral causes for AIDS.” The utility of the article as a historical summary of dissenting theories of AIDS was recognized by the reviewers and editors, who accepted the article for publication. Within days, several formal complaints were received by our office, and, in accordance with our complaints protocol, we submitted these to a group of Editors-in-Chief for their expert opinion. Based on their advice, Frontiers took three actions:

1. The article-type was changed to “Opinion” to better reflect the subjectivity of the subject matter and to clarify to the scientific community and broader readership that the work was not one of empirical basis.
2. Most importantly, several invited critical commentaries were published and linked directly to the published opinion article. These commentaries situated the original paper within the context of unsupported, fringe theories on HIV-AIDS. They were intended to ensure that all readers understand that the causal link between HIV and AIDS cannot be called into question.
3. Frontiers published a statement to clarify our decision concerning the above two points.

At the time, Frontiers intended that by classifying the publication as an “Opinion” with the critical commentaries would offset the potential risk that AIDS denialists would misrepresent the publication as scientific evidence to support their discredited claims. These efforts proved to be both misguided and ineffectual. The critical commentaries went largely unheeded while the original paper continued to attract attention. Since publication in September 2014, the paper has generated more than 91,800 views and continues to be shared on social media. By contrast, the two critical commentaries have generated less than 19,000 views between them.

In September 2018, Frontiers appointed a new Field Chief Editor to *Frontiers in Public Health*, Dr. Paolo Vineis of Imperial College, London. Since then, we have received new complaints, and we reopened the evaluation of the publication of Dr. Goodson's article. Our conclusion today is that the continued attention that this article garners of which almost none consider the necessary context provided in the critical commentaries itself presents a potential public health risk by lending credibility to refuted claims that place doubt on the HIV causation of AIDS. For these reasons, the editorial office of Frontiers and Field Chief Editor of *Frontiers in Public Health* retract this article. The author disagrees with the retraction.

